# Chronic *Trichuris muris* infection causes neoplastic change in the intestine and exacerbates tumour formation in APC min/+ mice

**DOI:** 10.1371/journal.pntd.0005708

**Published:** 2017-06-26

**Authors:** Kelly S. Hayes, Laura J. Cliffe, Alison J. Bancroft, Simon P. Forman, Seona Thompson, Cath Booth, Richard K. Grencis

**Affiliations:** 1 School of Biological Sciences, FBMH, MAHSC, University of Manchester, Manchester, United Kingdom; 2 Wellcome Trust Centre for Cell Matrix Research, University of Manchester, Manchester, United Kingdom; 3 Epistem Limited, Incubator Building, Manchester, United Kingdom; Queen's University Belfast, UNITED KINGDOM

## Abstract

Incidences of infection-related cancers are on the rise in developing countries where the prevalence of intestinal nematode worm infections are also high. *Trichuris muris* (*T*. *muris*) is a murine gut-dwelling nematode that is the direct model for human *T*. *trichiura*, one of the major soil-transmitted helminth infections of humans. In order to assess whether chronic infection with *T*. *muris* does indeed influence the development of cancer hallmarks, both wild type mice and colon cancer model (APC ^min/+^) mice were infected with this parasite. Parasite infection in wild type mice led to the development of neoplastic change similar to that seen in mice that had been treated with the carcinogen azoxymethane. Additionally, both chronic and acute infection in the APC^min/+^ mice led to an enhanced tumour development that was distinct to the site of infection suggesting systemic control. By blocking the parasite induced T regulatory response in these mice, the increase in the number of tumours following infection was abrogated. Thus *T*. *muris* infection alone causes an increase in gut pathologies that are known to be markers of cancer but also increases the incidence of tumour formation in a colon cancer model. The influence of parasitic worm infection on the development of cancer may therefore be significant.

## Author summary

It is estimated that now 2 billion people currently live with chronic parasitic worm infections. As the incidences of cancer increase worldwide, the importance of these chronic inflammatory conditions on the development of cancer becomes more important. Several bacterial, viral and parasitic infections are already known to influence cancer development but as colon cancer is particularly prevalent worldwide, we wanted to assess the effect of a large intestinal dwelling worm, *Trichuris muris (T*. *muris)* on its aetiology. This whipworm is a natural infection of mice and has significant homology to human whipworm. From our studies, we showed that chronic infection alone induced changes in the caecum of the mouse that were comparable to those seen with a well-known carcinogen. In addition to this, *T*. *muris* infection was also able to increase the development of adenomas in the small intestine of mutant mice that spontaneously develop tumours. This change was abrogated if a T regulatory cell type was blocked during infection. The T regulatory cell type that arises during infection has been shown to play an important role in protecting the host from damage caused by the parasite and the immune response to it. The present study using the mouse model however, suggests that regulatory T cells can have negative effects, at least in terms of the development of bowel cancer. As so many people live with chronic, regulated parasitic infections, the importance of the parasites in cancer development may therefore be significant.

## Introduction

Colon cancer is one of the leading causes of death within the western world and prevalence in developing countries has increased in the last decade [1]. There exists a strong link between inflammation and cancer [2]. This is emphasized in the colon where individuals with inflammatory bowel disease are predisposed to the development of colorectal cancer [3–5]. Furthermore, chronic infection and the resultant long-term exposure to inflammatory stimuli heighten the risk of neoplastic change. A number of chronic bacterial, viral and parasitic infections are associated with predisposition to neoplasia; *Helicobacter pylori* is associated with gastric cancer [6], Hepatitis B and C with liver cancer [7], *Clonorchis sinensis* with cholangiocarcinoma [8] and *Schistosome* infection with bladder cancer incidence [9].

Gastrointestinal worms, comprising *Ascaris lumbricoides*, *Trichuris trichiura*, *Necator americanus* and *Ancylostoma duodenale* species infect over 2 billion people worldwide and account for considerable morbidity and a loss of 5.2 million DALYS [10–12]. Individuals in endemic areas build up chronic infections due to repeated exposure with few people completely resolving infection. This chronic insult on the intestine is associated with intestinal inflammatory changes and it is now well understood that gut dwelling nematodes can manipulate the immune system (reviewed in [13,14]). Indeed, the therapeutic potential of worms in IBD [15,16], allergy [17,18] and inflammatory disease are apparent [19].

*Trichuris muris* (*T*. *muris*) is a natural parasite of mice and is extensively utilised as a laboratory model for the study of human whipworm infection, *T*. *trichiura* [20,21]. In susceptible hosts, the persistence of *T*. *muris* in the large intestine is characterised by the development of a strong type 1 (Th1) response, dysregulation of epithelial homeostasis and upregulation of inflammatory cytokines [22,23]. The generation of crypt cell hyperplasia is driven by an expansion of the proliferative compartment of the intestinal epithelium and is under immune control. The intestinal pathology associated with chronic *T*. *muris* infection closely resembles that seen in Crohn’s disease in humans and in human trichuriasis and is under the control of the regulatory cytokine IL-10 [24,25]. Given the heightened risk of IBD patients to colorectal carcinoma due to intestinal dysplasia and other genetic factors [26] and the global prevalence of intestinal helminth infection, it is clear that the nematode-neoplasia link warrants investigation. Here we assess the effects of a natural model of chronic intestinal helminth infection on the development of intestinal neoplasia.

## Materials and methods

### Ethics statement

Experiments were performed under the regulations of the Home Office Scientific Procedures Act (1986), Project licence 70/8127 and subject to review by the University of Manchester Animal Welfare and Ethical Review Body (AWERB). The experiments conform to the ARRIVE guidelines.

### Animals

Male wild type (WT) C57BL/6 mice aged 6–8 weeks were purchased from Envigo, U.K. APC^min/+^ mice were obtained from the Paterson Institute, Christie Hospital, Manchester, U.K. for initial d42 post infection (p.i.) studies, then from Birmingham University for all subsequent studies. C57BL/6 animals were used at 6–8 weeks of age and were housed for 7 days prior to experimentation. Both sexes of APC^min/+^ C57BL/6 mice were housed in the same facilities and infected at 12 weeks of age with group sizes of 8–12 mice. All animals were euthanized using a rising concentration of CO_2_.

### In vivo treatment

#### Azoxymethane (AOM) (Sigma, UK)

Animals were treated at the time of infection (day 0) and on day 7 p.i. with either 12.5mg/ml (adjusted for body weight) of AOM or vehicle control in 200μl of PBS by intraperitoneal (i.p.) injection.

#### Depletion of T regulatory (Treg) cells

Animals were treated with 500μg anti-CD25 antibody or Rat IgG1 isotype control (BioXCell, West Lebanon, USA) in 200μl of PBS by i.p. injection on days -4, 0, 7 and 14 p.i.

### Parasite and parasite antigen

#### T. muris

Stock infections of *T*. *muris* E isolate, originally obtained from The Wellcome Research Laboraties, Beckenham, Kent, were maintained in susceptible mouse strains (AKR, SCIDs or athymic). At day 42 p.i., the mice were killed and the caecum and adjacent colon removed. Worms were pulled out with fine forceps and cultured in RPMI-1640 (Gibco, UK) containing 2% FCS, 500 IU/ml penicillin, 500 μg/ml streptomycin and 2mM L-glutamine (all from Gibco, UK). 4 hour and overnight (O/N) culture supernatants containing both eggs and excretory/secretory products (E/S) were centrifuged at 720xg for 10 minutes. Egg pellets were washed in sterile water and centrifuged at 720xg for 5 minutes then filtered through a 100 μm nylon strainer (Falcon, USA) to remove residual worms and other contaminating debris. Eggs were placed in culture flasks (Helena Biosciences, UK) in distilled water and stored at room temperature in the dark for 8 weeks to allow embryonation.

Mice were infected by oral gavage, giving 20 embryonated eggs in ddH_2_O. Worm burdens were assessed at various time points p.i. To determine the level of infection in experimental animals, the caecum and adjacent colon of each mouse was removed and slit open longitudinally. For d18 p.i., the intestines were scraped with curved forceps to remove any embedded worms. Worms were then counted under a binocular dissecting microscope. D42 p.i. adult worms were counted by removing them individually with fine forceps.

Adult parasite antigen was obtained as follows; 4 hour and O/N culture supernatants were centrifuged at 720xg for 10 minutes, supernatants was filtered through a 0.22 μm filter (Millipore, UK), concentrated in a Centriprep concentrator (Amicon, UK) and dialysed against PBS overnight. E/S was again filtered though a 0.22 μm filter and the protein concentration was determined using a Nanodrop 100 (Labtech Ltd, UK). E/S was aliquoted and stored at—80°C. 4 hour E/S was used for *in vitro* restimulation of MLN cells and O/N E/S was used in parasite-specific antibody ELISA.

#### Heligmosomoides polygyrus (H. polygyrus)

Stock infections of *H*. *polygyrus* were maintained as follows. Susceptible mice were infected with 300 L3 larvae by oral gavage and maintained for up to 28 days. Faecal pellets from infected mice were collected from day 10 post-infection and plated with activated charcoal onto filter paper (Whatmann No1 circles). Samples were incubated for 9 days at room temperature to allow eggs to hatch and develop to the infective larval (L3) stage. Larvae were collected from plates and washed 3 times in water and stored for up to 60 days at 4°C before use. To determine the level of infection at day 28 p.i., adult worms were collected using the Baermann technique and counted using a binocular dissecting microscope.

For the generation of antigen for *in vitro* mesenteric lymph node (MLN) restimulations, adult worms were obtained as described above and homogenized in a pestle and mortar at 4°C until no whole worms could be observed under a dissecting microscope. The homogenate was left for 1 hour at 4°C before centrifugation at 15,700xg for 20 minutes. The supernatant was filtered through a 0.22mm sieve (Millipore corporation) and dialyzed for 24 hours in 3 changes of 5L PBS at 4°C. Protein concentration was determined by Nanodrop 100 (Labtech Ltd, UK).

### Cell culture and cytokine analysis

MLN and spleen cells were removed, cultured and restimulated for 24 hours under conditions previously described [27].We measured concentrations of TNF-α, IFN-γ, IL-6 and IL-10 in the culture supernatants using a cytokine bead assay (CBA, BD Biosciences, UK) performed according to the manufacturer’s instructions.

### Parasite specific antibody analysis

ELISA plates were coated with 5 μg/ml of overnight E/S in 0.05 M carbonate/bicarbonate buffer, pH 9.6 and incubated overnight at 4°C. Plates were blocked for 1 hour with 150 μl PBS/Tween-20 (PBST), 3% BSA at room temperature. Eight serial two-fold dilutions of sera in PBST were conducted from 1/20 to 1/2560 and transferred to the ELISA plates (50 μl/well) for 90 minutes at room temperature. Parasite specific IgG1 and IgG2a were detected using biotinylated rat-anti mouse antibodies (Pharmingen, UK and Serotec, UK respectively) diluted in PBST, 50 μl/well for 1 hour at room temperature. Streptavidin peroxidase was added at 75 μl/well for 1 hour and ABTS substrate was added at 100 μl/well. Plates were read after approximately 20 minutes at 405nm on a VersaMax microplate reader (Molecular devices, UK).

### Fluorescence-activated cell sorting (FACS)

Proportions of CD4+, CD25+ and FoxP3+ cells in the MLN and spleen of WT animals were analysed using flow cytometry at day 80 p.i. FITC anti-CD3(ε) in combination with streptavidin-allophycocyanin, (Caltag Laboratories, Burlingame, CA) FoxP3 and a CD25 FL3 (PharMingen) were used for surface marker staining. Cells were analysed using CellQuest Pro software (BD Biosciences, UK). In subsequent APC^min*/+*^ mice experiments, cells were analysed on a MACSQuant (Miltenyi Biotec, UK) using FITC-CD4, PE-CD25 and APC-FoxP3 (BD Biosciences, UK).

### Tumour burden in APC^min/+^ mice

Both small and large intestine were removed from animals at autopsy, gently flushed out using saline, slit longitudinally and cut into 2 cm pieces. Sections were pinned out on wax coated petri dishes with the luminal face of the intestine facing upwards, and fixed with 4% formaldehyde for 24 hours. Sections of intestine were stained whilst pinned out with methylene blue to allow the visualisation of tumours (5 minutes at room temperature). For regional analysis of tumour burden in the small intestine, the intestine was divided into 3 equal length sections-from the duodenum to the ileum and labelled SA to SC respectively. Within each region, the intestine was divided into 2 cm pieces, which were assessed for tumour burden. The area of tissue, number and size of tumours was determined using a computer assisted Zeiss Axiohome^™^ microscope system under x 40 magnification.

### Tissue preparation for detection of apoptosis, proliferation and neoplastic change

Samples of cecum were removed and flushed out using saline. Samples were fixed intact in carnoy’s fixative for 30 minutes prior to storage in 70% ethanol. Tissues were prepared using the gut bundle technique [28]. Tissues were then paraffin embedded using standard histological techniques and 3μm sections were cut.

### Detection of apoptotic cells

Sections were stained with haematoxylin and eosin (H&E) to allow the visualisation of apoptotic cells. Such cells are detected on the basis of their morphology using light microscopy, a method that has been used extensively [29–32]. Typically, apoptotic cells appear pink, circular, with crescent shaped nucleus, and are bubbled up out of the plane of focus. TUNEL labelling is another method which has can be used to detect apoptosis in the intestinal epithelium. However, this technique is prone to false positive and false negative results when compared with morphological assessment, as well as failing to distinguish between DNA cleaved by apoptosis and DNA fragments cleaved by other processes [33]. For the purpose of this investigation, therefore, morphological analysis was deemed the most reliable method to use.

### Epithelial cell proliferation

Groups of 4 mice were treated by i.p. injection with 10mg BrdU (Sigma, Poole, U.K.) 40 minutes prior to sacrifice. All animals were killed at the same time- within and between experiments to minimise any differences in proliferation attributable to variation in circadian rhythm. Detection of nuclei that had incorporated BrdU was performed by immunohistochemistry, using a monoclonal anti-BrdU antibody (Mas 250b, Harlan Sera Laboratories, Loughborough, U.K.) as described [34]. Sections were analysed by scoring 50 caecal crypts per mouse, 4 mice per group.

### Scoring of epithelial cell apoptosis and proliferation

Full-length longitudinal sections of crypts were selected for analysis. The blinded scoring commenced with the cell at the mid-point at the base of the crypt, which was designated as position 1 and continued until the crypt-crypt table was reached. This method of scoring allows the generation of statistically valid results [34] and was used to determine the levels of apoptotic and proliferating cells. In this way both the position and overall numbers of apoptopic or proliferating cells in the cecum can be determined.

### Measurement of crypt length, width and epithelial area

Area of epithelium was assessed using the computer assisted Zeiss Axiohome^™^ Microscope system to mark around the area of interest. Analysis was performed on H&E stained sections, 4 mice per group, 3–4 circumferences per mouse. Circumference of the lumen was subtracted from circumference of the muscularis to give the area of epithelium. Individual crypt length and widths were determined using the same system, selecting well-orientated crypts and measuring from the base of the crypt to the lumen for crypt length, and the widest area of the crypt for width analysis. 50 crypts per mouse were measured, 4 mice per group.

### Detection of aberrant crypt foci/neoplastic change

Aberrant crypts were detected on the basis of their morphology on H&E stained cross sections of intestine as described [35]. A scoring system was devised to assess the severity of aberrant crypts, aberrant crypt foci [multiple aberrant crypts], epithelial hyperplasia and adenoma formation. Scores were assigned on the basis of number of aberrant crypts, the number aberrant crypt foci (clusters) per circumference as well as the degree of area of epithelium affected. A score of 0 indicates no detectable change and 4 the highest level of severity.

### Statistical analysis

Statistical analysis was performed using Students t test. A value of p<0.05 was considered to be significant.

## Results

**Fig 1:**
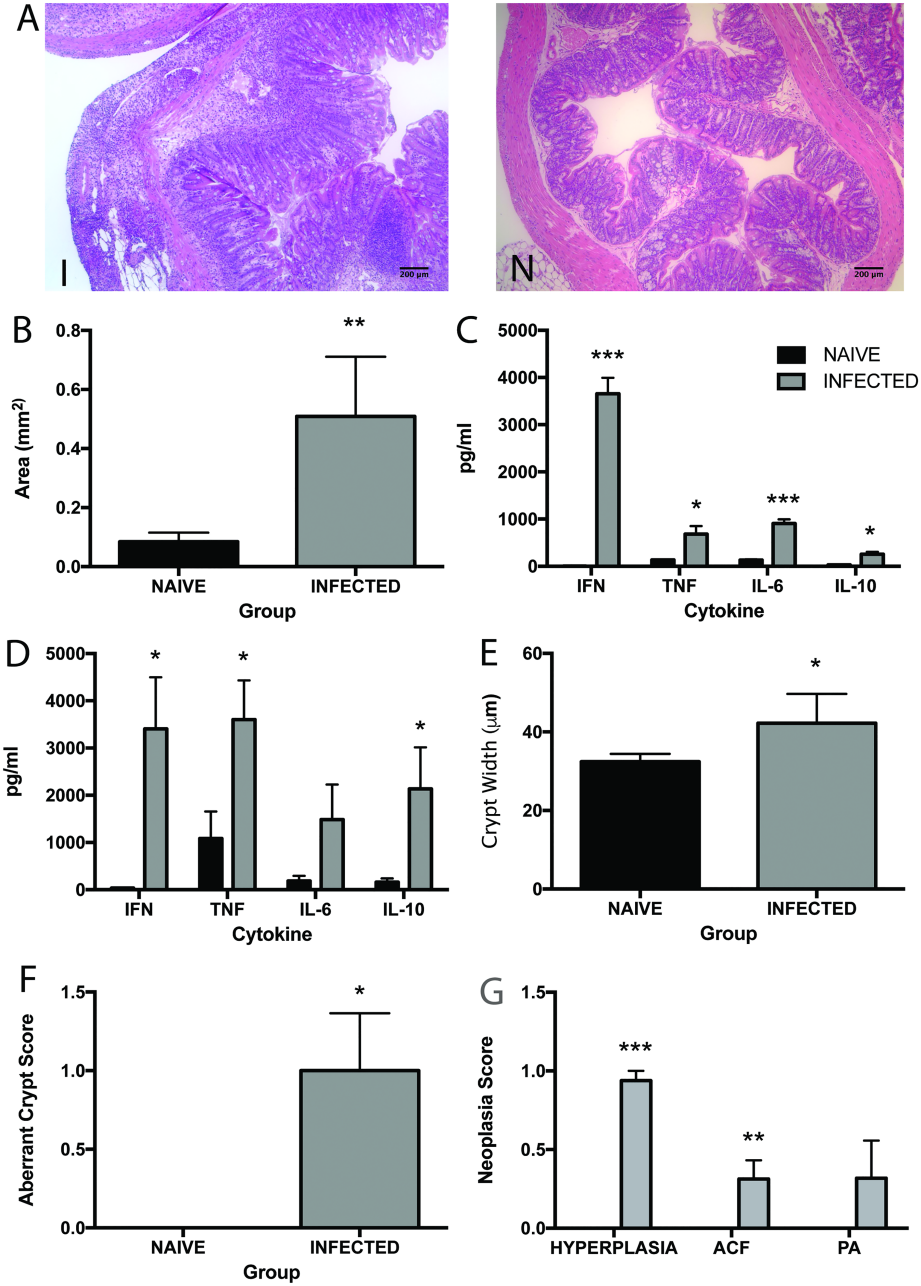
Chronic *T*. *muris* infection induces intestinal inflammation and hallmarks of neoplastic change. C57BL/6 animals at day 80 p.i. **A** H&E stained caecal sections from a naïve (N) and infected (I) animal. **B** Total area of lamina propria and epithelium in the intestine **C** IL-6, IFN-γ, TNF-α and IL-10 produced by re-stimulated MLN cells. **D** IL-6, IFN-γ, TNF-α and IL-10 produced by re-stimulated spleen cells. **E** Crypt width in the caecum and colon. **F** Aberrant crypt score in caecum and colon. **G** Neoplasia score based on extent of hyperplasia, numbers of aberrant crypt foci (clusters) and numbers of pre-adenomas in caecum and colon. Black bars denote naïve animals and grey bars denote infected animals. * significant difference between naïve and infected animals p<0.05, ** p<0.005 and *** p<0.0005. n = 4–8 per group.

WT C57BL/6 mice develop a regulated Th1 driven intestinal inflammation during chronic infection with *T*. *muris* [36]. Given the strong association with the prolonged exposure to pro-inflammatory cytokines and the development of neoplasia, we were interested to see whether chronic infection was associated with neoplastic change in the intestine of WT mice during chronic infection. Gut pathology was assessed in WT C57BL/6 mice at day 80 p.i. This time point was selected as it ensured that animals had been exposed to inflammation for a long time period. An increase in lamina propria cell infiltrate was detected in infected animals over naive as well as epithelial hyperplasia (Fig 1A & 1B). Assessment of the levels of pro-inflammatory cytokine production revealed that the levels of IL-6, TNF-α and IFN-γ were increased in both the MLN (Fig 1C) and spleen (Fig 1D) at day 80 p.i. Chronic *T*. *muris* infection is also known to induce an anti-inflammatory IL-10 response in the host that acts to regulate pathology [25,37,38]. Interestingly, although the levels of the IL-10 increased significantly (p<0.05) in the spleen the levels were only minimally increased in the draining lymph node at the site of infection as reflected by antigen specific recall stimulation of cells from the MLN (Fig 1C & 1D).

Gut dysplasia was assessed using a number of histological markers, including the presence of epithelial cell hyperplasia, measurement of crypt width and the number of both aberrant crypts and aberrant crypt foci (clusters of aberrant crypts). Aberrant crypt foci were first described as lesions consisting of thick irregular (aberrant) crypts in methylene blue stained sections detectable following carcinogen treatment of mice [35,39]. These lesions have since been found to correlate with the degree of colonic neoplasia in humans [40,41], and are hypothesized to serve as biomarkers for colorectal adenoma and cancers [41]. At day 80 p.i. there was a significant increase in the development of crypt width and aberrant crypt formation in infected animals over naïve (Fig 1E & 1F). Additionally, the neoplasia score was increased in infected animals in all 3 categories, i.e. extent of hyperplasia, number of aberrant crypt foci and number of pre-adenomas (Fig 1G).

**Fig 2:**
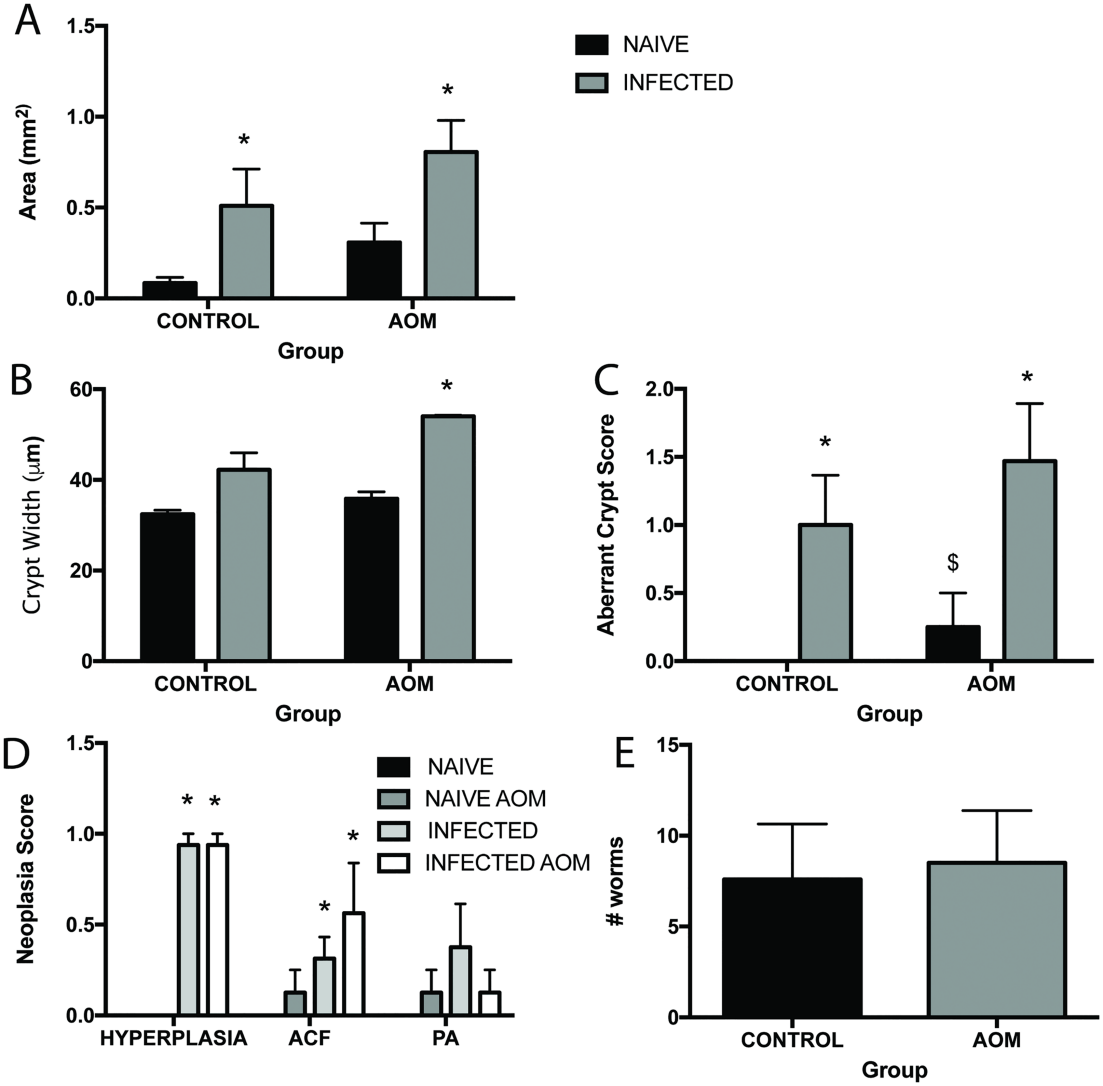
Chronic *T*. *muris* infection exasperates epithelial neoplasia in the colon carcinoma AOM model. C57BL/6 mice at day 80 p.i. treated with AOM or PBS control. **A** Total area of lamina propria and epithelium in the intestine. **B** Crypt width in the caecum and colon. **C** Aberrant crypt score in caecum and colon. **D** Neoplasia score based on extent of hyperplasia, numbers of aberrant crypt foci (clusters) and numbers of pre-adenomas in caecum and colon. Black bars denote naïve animals and grey bars denote infected animals. **E** Numbers of worms in control (black bars) and AOM (grey bars) treated mice at day 80 p.i. * significant difference between naïve and infected animals p<0.05, $ significant difference between naïve animals in PBS and AOM groups p<0.05. n = 4–8 per group.

In order to determine the extent of neoplastic change caused by chronic *T*. *muris* infection, the effects seen with infection were compared to those seen when mice had been treated with a carcinogen. AOM is routinely used as a colon specific genotoxic carcinogen [42], therefore animals were treated with AOM at day 0 and 7 p.i. and infection was allowed to progress to chronicity. Using the histological markers for inflammation and neoplastic change used before, it can be seen that changes observed following infection (grey bars in PBS group) were as marked or greater (in the case of aberrant crypt score) as those seen with AOM treatment (black bar in AOM group) (Fig 2A–2D). Interestingly, AOM treatment had no effect on the worm burdens of mice (Fig 2E) and AOM-infected mice had similar histology scores in terms of crypt width, aberrant crypt score and neoplasia score to PBS-infected mice (Fig 2B–2D). AOM-infected groups however had significantly increased crypt width, aberrant crypt score and neoplasia scores when compared to AOM-naïve groups (Fig 2B–2D) further demonstrating the marked changes that *T*. *muris* is causing within the intestine. Cytokine production in the MLN of infected animals were similar between groups (S1 Fig) though, interestingly, naïve AOM treated animals produced increased amounts of the proinflammatory cytokines IFN-γ, TNF-α and IL-6.

As *T*. *muris* infection is clearly associated with neoplastic change in the large intestine and exacerbated compared to that seen with AOM treatment, we were interested to see whether *T*. *muris* had any effect on spontaneous adenoma formation in the well-established model of intestinal neoplasia, the APC^min/+^ (Adenomatosis Polyposis Coli) mouse. Both humans and mice with a germ-line mutation in the APC gene have a predisposition to intestinal neoplasia [43]. In mice, *Min* (multiple intestinal neoplasia) is a dominant trait involving a nonsense mutation in codon 850 of the APC homologue. Loss of APC heterozygosity in these animals results in adenoma formation through out the GI tract [44]. APC^min/+^ mice are routinely used as model for human Familial Adenomatous Polyposis (FAP). Although there is a clear genetic basis for adenoma formation in these animals, tumorigenesis is also influenced by a number of environmental factors, including bacterial infection, inflammation and toxic insult [45,46].

**Fig 3:**
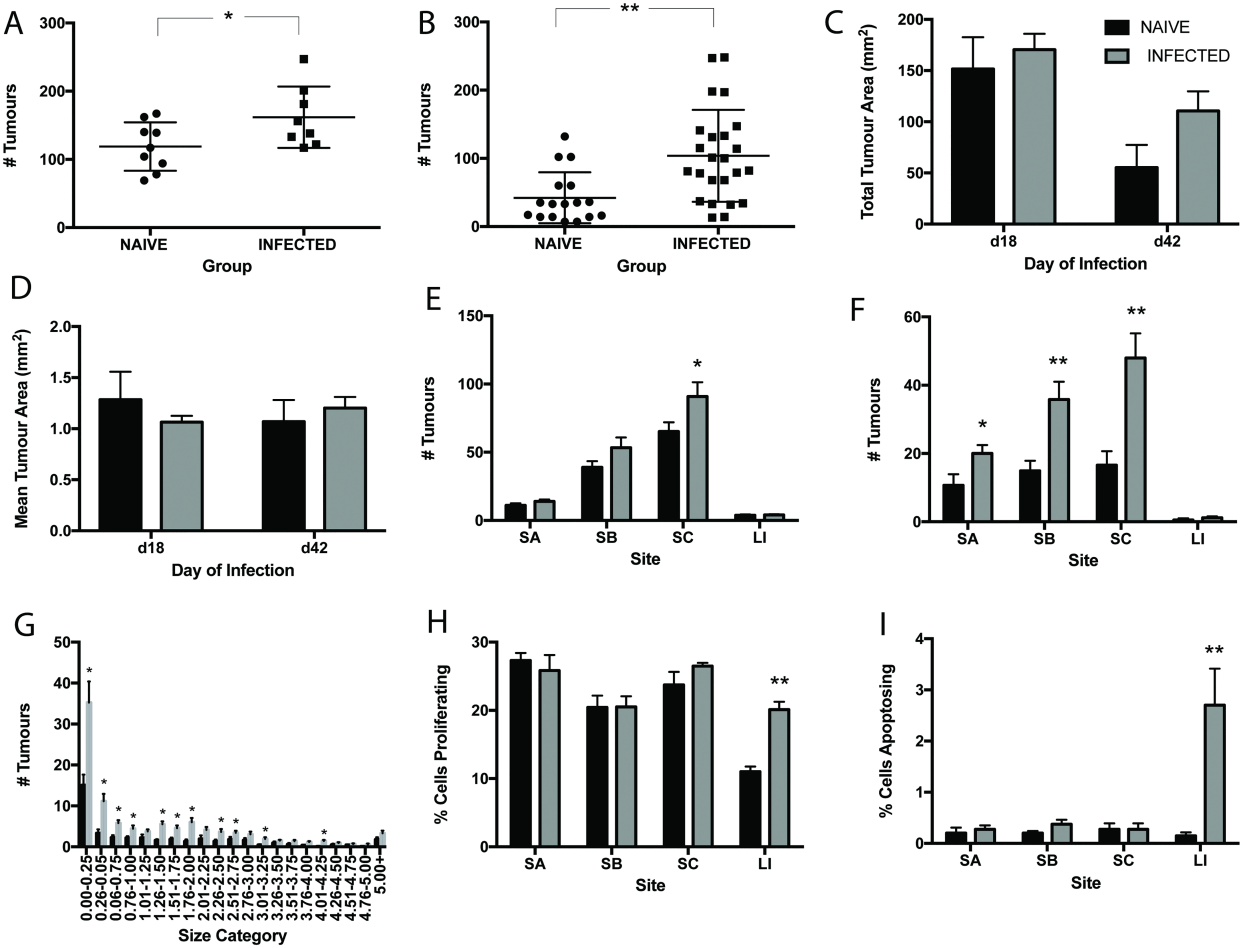
Chronic *T*. *muris* infection promotes the development of intestinal neoplasia in APC^min/+^ mice. *T*. *muris* infection at day 18 p.i. and day 42 p.i. in APC^min/+^ mice. **A** Total number of tumours in the intestines of naïve and infected mice at day 18 p.i. **B** Total number of tumours in intestines of naïve and infected mice at day 42 p.i. **C** Total tumour area in intestines of naïve and infected mice at d18 and d42 p.i. **D** Mean tumour area in intestines of naïve and infected mice at d18 and d42 p.i. **E** Number of tumours in regions of the intestine in naïve and infected mice at day 18 p.i. SA upper, SB mid, SC lower small intestine and LI large intestine. **F** Number of tumours in regions of the intestine in naïve and infected mice at day 42 p.i. **G** Size frequency distribution of tumour size between naïve and infected animals at day 42 p.i. **H** Percentage of proliferating cells in regions of the intestine at day 42 p.i. **I** Percentage of apoptopic cells in regions of the intestine at day 42 p.i. Black bars denote naïve and grey bars denote infected animals, * significant difference between naïve and infected animals p<0.05, ** p<0.005. APC^min/+^ mice from the Paterson Institute, Manchester UK. n = 8–13 per group and repeated twice.

Mutant mice were given a low level *T*. *muris* infection to ensure chronicity and to mirror the typical parasite burden of naturally infected individuals. Patent infection developed in APC^min*/+*^ mice, characterized by a Th1 dominated immune response and high levels of parasite specific IgG2a (S2 Fig). Animals were infected with *T*. *muris* at 12 weeks of age. This age was chosen, as this is the time when adenomas start to form in the colony of APC^min/+^ animals used. It is important to note that both the onset and severity of adenoma formation varies between colonies of APC^min/+^ mice and that for practical reasons, different colonies were used for day 18 and day 42 studies. In order to minimize variability between experiments, animals were housed in the same facility during experimentation and were age-matched. Analysis of tumour burden at 42 p.i. revealed that persistent *T*. *muris* did not promote tumour formation in the large intestine, the niche of the parasite (number of tumours in naïve animals 3.07±0.51 as compared to in infected animals 3.00±0.44). Surprisingly, *T*. *muris* infection did promote the development of adenoma formation throughout the small intestine. The number of tumours throughout the intestine was significantly increased at both day 18 and day 42 p.i. (Fig 3A & 3B). Total tumour area and mean tumour area however, were unchanged upon infection (Fig 3C & 3D). Enhanced adenoma formation in the small intestine was greatest in the lower small intestine (SC) (2.5 fold increase at day 42 p.i.), in comparison with the upper small intestine (SA). These changes were apparent at day 18 p.i. but more distinct by d42 p.i. (Fig 3E & 3F). Although no differences in total size and mean size of tumours was apparent on infection, there was a significant increase in the number of small tumours upon infection (Fig 3G).

It is clear from previous investigation that *T*. *muris* induces homeostatic dysregulation in the gut [22,23,47]. In an attempt to determine whether worm induced perturbation in epithelial cell cycle was playing a role in tumorgenesis in APC^min/+^ mice, the levels of cell proliferation and apoptosis were assessed throughout the intestine. Both cell proliferation (Fig 3H) and cell death (Fig 3I) increased in the large intestine during chronic infection. An increase in the number of aopotopic cells was observed at the base of the crypts whilst more proliferating cells were found further up the crypt axis in infected animals. However, no effect of infection upon proliferation or apoptosis in the small intestine was seen despite its effect on adenoma formation at this site.

**Fig 4:**
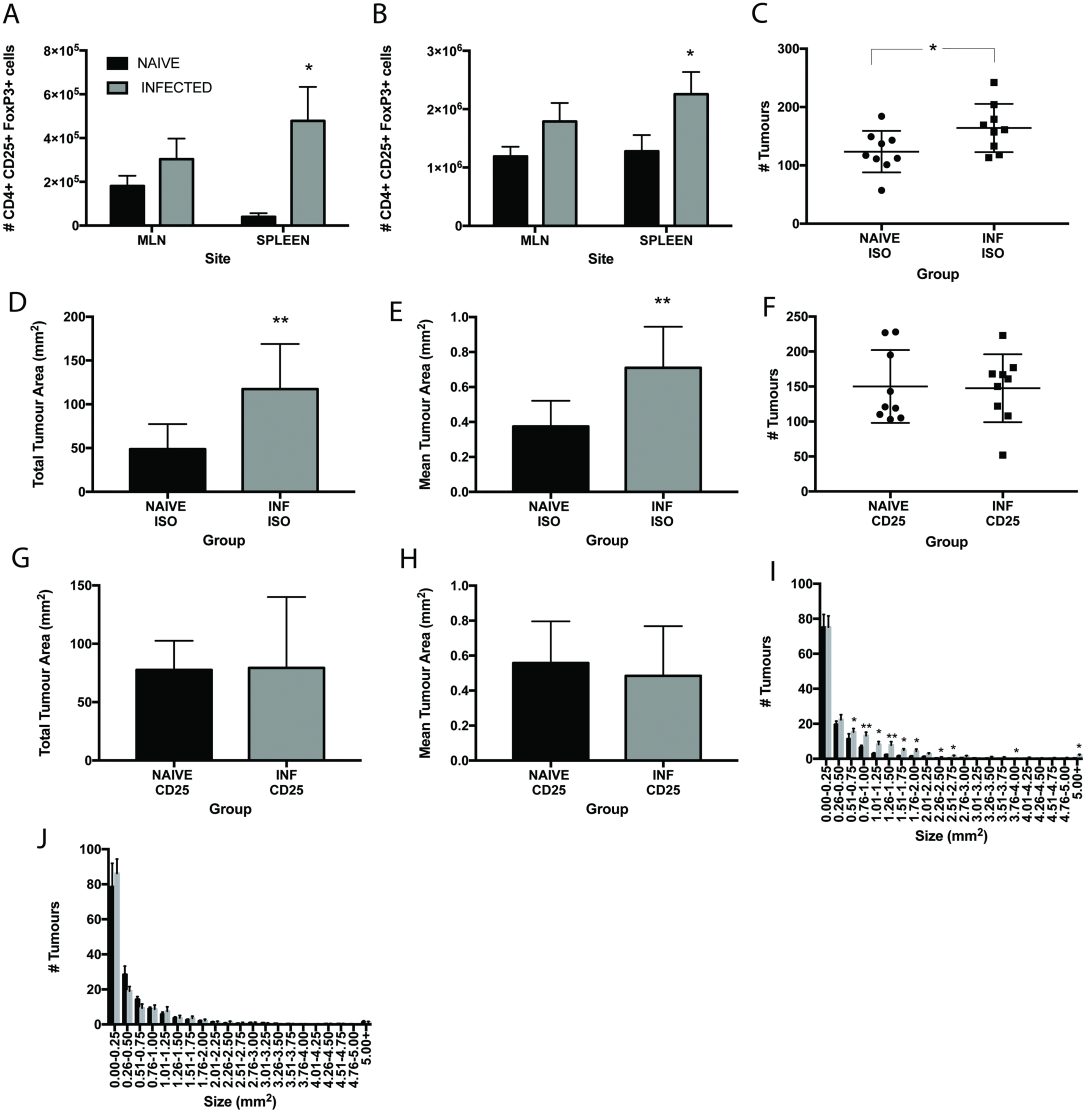
Anti-CD25 treatment of *T*. *muris* infected APC^min/+^ mice abrogates the increase in tumour formation seen in isotype treated animals. *T*. *muris* infected APC^min/+^ mice at day 18 p.i. treated with isotype control or anti-CD25 antibody during infection. **A** Numbers of CD4+CD25+FoxP3+ cells in the MLN and spleen of naïve and chronically infected WT C57BL/6 mice at day 80 p.i. **B** Numbers of CD4+CD25+FoxP3+ cells in the MLN and spleen of naïve and chronically infected APC^min/+^ mice at day 18 p.i. **C** Total number of tumours in the intestine in naïve and infected APC^min/+^ mice at day 18p.i. in control isotype treated groups. **D** Total tumour area in intestine of naïve and infected APC^min/+^ mice at d18p.i in control isotype treated groups. **E** Mean tumour area in intestine tract of naïve and infected APC^min/+^ mice at d18 in control isotype treated groups. **F** Total number of tumours in the intestine in naïve and infected APC^min/+^ mice at day 18p.i. in anti-CD25 treated groups. **G** Total tumour area in intestine of naïve and infected APC^min/+^ mice at d18 p.i. in anti-CD25 treated groups. **H** Mean tumour area in intestine tract of naïve and infected APC^min/+^ mice at d18 p.i. in anti-CD25 treated groups. **I** Size frequency distribution of tumour size between isotype treated naïve and infected APC^min/+^ mice. **J** Size frequency distribution of tumour size between anti-CD25 treated naïve and infected APC^min/+^ mice. Black bars denote naïve animals and grey bars denote infected animals. * significant difference between naïve and infected animals p<0.05, ** p<0.005. APC^min/+^ mice from Birmingham University, UK. n = 8–9 per group.

Chronic *T*. *muris* infection is known to drive a Treg response in mice [48]. Indeed an increase in the numbers of CD4+CD25+FoxP3+ cells is evident in the spleen of chronically infected mice (Fig 4A) and at day 18p.i. in APC^min*/+*^ mice (Fig 4B). A strong correlation between the presence of Tregs and the inhibition of tumour immunosurveillance has been reported in a number of cancers (reviewed in [49]). To address whether Tregs were playing a role in the promotion of neoplasia in this study, APC^min/+^ mice were treated with anti-CD25 monoclonal antibody throughout infection and the effects assessed at day 18 p.i. Antibody treatment significantly reduced the numbers of CD4+CD25+FoxP3+ cells in the MLN and spleen of treated animals (S3 Fig). As shown previously, numbers of tumours were increased upon infection in control isotype treated animals (Fig 4C). Moreover, in this experiment and cohort of APC^min/+^ mice, an increase in the total tumour area (Fig 4D) and mean tumour area (Fig 4E) was seen upon infection in the isotype treated groups. Interestingly, anti-CD25 treatment increased the tumour number and area in naïve mice as compared to isotype treated animals although this was not significant. There was no change upon infection in number, total or mean tumour area in the anti-CD25 treated animals (Fig 4F–4H). This was further confirmed when assessing tumour size. Isotype treated mice showed a significant increase in the smaller sizes of tumours upon infection (Fig 4I) whilst there were no differences in any size category between anti-CD25 treated naïve and infected animals (Fig 4J). Importantly, antibody treatment did not affect worm burden (S2 Fig). Thus depression of CD4+CD25+FoxP3+ numbers was associated with the capacity to control tumours in the gastrointestinal tract of *T*. *muris* infected mice.

**Fig 5:**
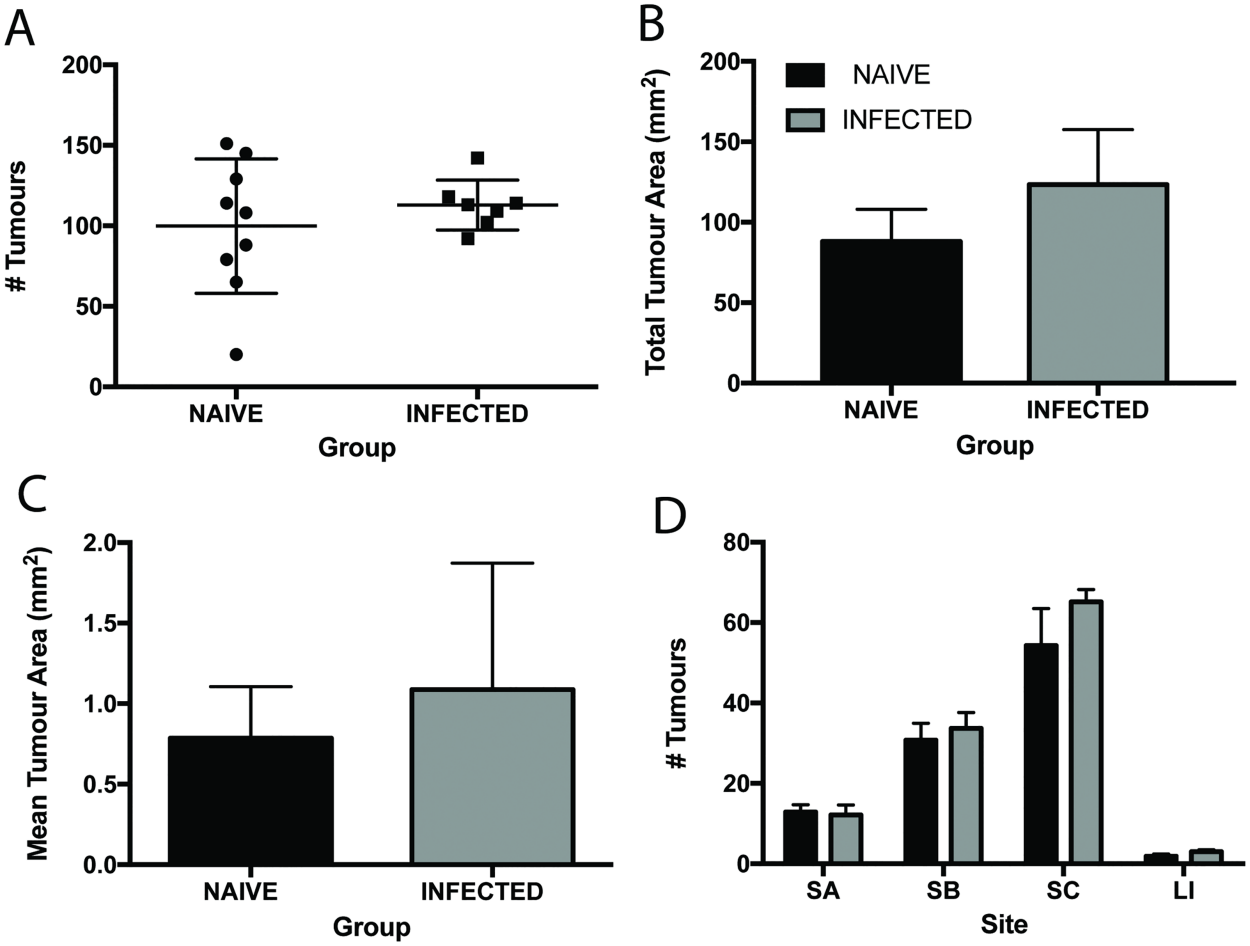
The effects of *T*. *muris* infection on intestinal neoplasia in APC^min/+^ are specific to *T*. *muris*. *H*. *polygyrus* infected APC^min/+^ mice at day 28 p.i. **A** Total numbers of tumours in the intestines of naïve and *H*. *polygyrus* infected mice. **B** Total tumour area in the intestines of naïve and *H*. *polygyrus* infected mice. **C** Mean tumour area in the intestines of naïve and *H*. *polygyrus* infected mice. **D** Number of tumours in regions of the intestine in naïve and infected mice at day 18 p.i. SA upper, SB mid, SC lower small intestine and LI large intestine. Black bars denote naïve animals and grey bars denote infected animals. APC^min/+^ mice from Birmingham University, UK. n = 7–9 per group.

In order to confirm if our observations for *T*. *muris* extend to other GI nematode parasites, we used a different parasitic worm, *Heligmosomoides polygyrus* (*H*. *polygyrus)*. This parasite resides in the small intestine of the mouse so also allows us to assess the effect of the physical damage caused by a large parasite on neoplastic change at the site of adenoma formation. APC^min/+^ mice were infected at 12 weeks of age and the effects of infection assessed on day 28 p.i., a time by which *T*. *muris* infected APC^min/+^ mice had significantly increased intestinal neoplastic change. All infected animals had multiple worms in the small intestine on autopsy. The numbers of Tregs were unchanged from naïve levels in both the MLN and spleen of infected mice (S3 Fig). In contrast to *T*. *muris* infection, there were no changes seen in tumour number, mean tumour area or total tumour area in *H*. *polygyrus* infected mice (Fig 5A–5C) at this time point. This was also apparent when examining the numbers of tumours in different locations of the small intestine, where no changes were seen with infection (Fig 5D).

### Conclusion

Here we demonstrate that low-level chronic *T*. *muris* infection promoted the development of intestinal neoplasia to a level that is comparable to that induced by a chemical carcinogen. Moreover, the observation that *T*. *muris* increased the neoplastic change seen in AOM treated mice and promoted tumour formation in APC^min/+^ mice identified that gut dwelling nematode infection can induce simultaneous activation of local and systemic dysplasia. We have also identified that the *T*. *muris* induced Treg response that accompanies infection may negatively influence the neoplastic change in WT mice and tumour development in APC^min/+^ mice.

Genetic changes such as activated oncogenes or altered tumour suppressor genes (such as APC) in tumour cells are responsible for many aspects of neoplasia, indeed, over 80% of colorectal cancer cases are proposed to be due to the loss of APC [50]. It is now established that an inflammatory environment also plays a role [2,51,52]. During chronic *T*. *muris* infection in C57BL/6 animals there is intestinal inflammation, a large influx of inflammatory cells into the intestine and elevated levels of pro-inflammatory cytokine production in the MLN and spleen (Fig 1A–1D). This infection-induced inflammation may be promoting the development of epithelial neoplasia in these mice (Fig 1E–1G) and indeed, other intestinal infections have been shown to promote tumour formation in an inflammation dependent manner [53]. In order to quantify the neoplastic change seen with infection we used the AOM model of intestinal cancer. *T*. *muris* infection induced an increase in aberrant crypt foci and in hyperplasia as compared to AOM alone (Fig 2C and 2D). However, infection and AOM in combination did not show additional changes over infection alone. Thus we can conclude that *T*. *muris* infection initiates neoplastic changes in the gut that are significantly increased when compared to those seen with a commonly used chemical carcinogen.

To assess the effect of *T*. *muris* on a model of spontaneous neoplastic change rather than chemical induced tumours, we used the APC^min/+^ mouse model of colon cancer. Interestingly, *T*. *muris*, a nematode that resides in the large intestine, was able to exacerbate intestinal neoplasia throughout the intestinal tract in these animals (Fig 3A–3F). This clearly demonstrates that a caecal nematode infection can potentiate neoplasia in both a localized and systemic manner. Even within 18 days of infection, significant changes were seen within the lower ileum and in the number of smaller tumours found. This progressed to significant changes seen throughout the small intestine by day 42 p.i. with even more size categories of tumours affected. The finding that the greatest increase in tumour number was in tumours of the smaller size category (Fig 3G) strongly suggested that *T*. *muris* infection was acting to promote new tumour formation rather than enhancing the growth of well differentiated preexisting tumours. There was no difference between mean tumour size in naïve and infected animals (Fig 3C & 3D), again supporting the hypothesis that infection does not significantly affect the growth of pre-existing tumours. *T*. *muris* is known to cause epithelial dysregulation in the intestine with increased epithelial proliferation and apoptosis [22,47], both mechanisms which could lead to tumour formation [54]. However, changes in these mechanisms were only found within the caecum, the parasite niche, and not in the small intestine, which is the site of most neoplastic change (Fig 3H & 3I). Therefore, although epithelial homeostasis may play an important role in the development of worm-induced dysplasia in the large intestine, it appears to have minimal impact in the small intestine.

Typical inflammatory cytokines associated with chronic *T*. *muris* infection were seen in both the MLN and spleen of infected APC^min/+^ mice (S2 Fig). This complements studies by Rao et al [53] that demonstrates that *H*. *hepaticus* infection promotes tumour development both locally in the intestine as well as systemically in mammary tissue in APC^min/+^ mice due to inflammatory cytokine production and studies on the cytokine microenvironment in these mice [55]. Furthermore, the administration of dextran sulphate sodium (DSS) to APC^min/+^ mice exacerbates adenoma formation, highlighting the importance of intestinal inflammation in promoting adenoma formation in this system [56] and the ablation of inflammatory cytokines leads to a decrease in adenomas [57]. Additionally, an increased pro-inflammatory cytokine production seen in *T*. *muris* infected mice over naïve mice treated with AOM alone may explain the increased neoplastic change (S1 Fig). However, a *T*. *muris* infection also promotes a robust Treg response that protects the host from damage [48]. Indeed, a key cytokine produced by Treg cells, IL-10, is critical in host survival during *T*. *muris* infection [37]. The successful use of *T*. *muris* to counter allergy and to protect against colitis in mouse models has been postulated to be due to this induced Treg response and its ability to immune modulate. In other systems, immune suppression can promote cancer through the down regulation of the anti-tumour immune response [reviewed in [49]] although paradoxically, in colon cancer Tregs are found to play a protective role [58–60] and this may be down to the type of Tregs that are found [61] or indeed the balance of cytokine production and Tregs [62].

Using anti-CD25 monoclonal antibody treatment to depress the number of Treg cells *in vivo* during the course of *T*. *muris* infection significantly reduced the number of CD4+CD25+FoxP3+ cells in the MLN and spleen of treated APC^min/+^ mice. In the isotype treated animals, infection increased the number of tumours (Fig 4C) in the mice as demonstrated previously (Fig 3A & 3B). However, the mean tumour area (Fig 4D) and total tumour area (Fig 4E) was also increased as compared to the previous study where it was unchanged after infection (Fig 3C & 3D) suggesting an effect of *T*. *muris* on the growth of the tumours rather than initiation. It is worthwhile to note that this colony developed a significantly higher number of tumours and showed clinical signs of disease much earlier than the previous colony suggesting an earlier advancement of the disease. This raises the interesting question of whether the timing of infection in the context of tumour development is important. It was clear however that *T*. *muris* infection still induced neoplastic change. In contrast, there were no differences in any neoplastic change readout between the infected and naïve groups of the anti-CD25 treated mice, supporting a role for Tregs in suppressing tumour control in infected mice. Anti-CD25 treatment of naïve animals did increase the numbers of tumours and the mean tumour area as compared to isotype treated animals suggesting a role for CD25+ cells in protection against spontaneous neoplastic change in the APC^min/+^ mouse. The role of Tregs in APC^min/+^ is complex and findings differ between studies [63–66]. This may in part be due to the phenotype of the T reg present and the microenvironment [61,62] and would certainly warrant further investigation in the context of *T*. *muris* infection. Regardless of the effects in naïve mice, the data here strongly supports a role for parasite-infection induced CD25+ T cells in suppressing anti-tumour immunity.

To confirm whether observations were specific to *T*. *muris* infection or a reflection of intestinal helminth infection in general, APC^min/+^ mice were infected with *H polygyrus*. *H*. *polygyrus* is a small intestinal dwelling parasite that presents as a chronic primary infection and at day 28 p.i. does not induce a marked CD4+CD25+FoxP3+ response (S3 Fig) [67]. This parasite model had the added benefit of allowing assessment of any neoplastic change as a result of mechanical damage by the worm at the site where neoplastic changes were evident. *H*. *polygyrus* did not induce any significant changes in any of the parameters assessed. Additionally *H*. *polygyrus* did not elicit as strong a proinflammatory environment in the MLN or spleen as observed with *T*. *muris* (S4 Fig). Whether a more prolonged infection would result in such changes requires further investigation.

The importance of the *T*. *mur*i*s* induced Treg response being detrimental, in terms of tumour control, to the host is important as the regulatory response induced by the parasite has been suggested to be beneficial by controlling colitis and allergy in mice [15–17]. We propose that this infection induced Treg response actually has detrimental consequences for both WT mice and APC^min/+^ mice. The promotion of neoplasia by *T*. *mur*is has important connotations given that 600 million people [11] harbor chronic infection with this genus. Ultimately, the impact of such infections warrant further investigation, particularly when considering the rising trend of cancer prevalence and, in particular, infection-induced cancers in developing countries [68,69].

## Supporting information

S1 Fig A IFN-γ, B IL-6, C TNF-α and D IL-10 produced by re-stimulated MLN cells from *T*. *muris* infected C57BL/6 mice at day 18 p.i. Black bars denote PBS treated animals and grey bars denote AOM treated animals. * p<0.05 ** p<0.005 ***p<0.0005 n = 4 per group.
(TIF)

S2 Fig **A** IFN-γ, **B** IL-6, **C** TNF-α and **D** IL-10 produced by re-stimulated MLN cells from naïve and *T*. *muris* infected APC^min/+^ mice at day 18 p.i. Black bars denote naïve animals and grey bars denote infected animals. **E** IgG2a production in naïve and *T*. *muris* infected APC^min/+^ mice serum at day 42 p.i. * p<0.05, *** p<0.0005 n = 4 per group.
(TIF)

S3 Fig **A** Number of CD4+CD25+FoxP3+ cells in MLN and **B** Spleen from isotype and anti-CD25 treated naïve and *T*. *muris* infected APC^min/+^ mice. C Worm burdens in isotype and anti-CD25 treated animals at day 18 p.i. Black bars denote naïve animals and grey bars denote infected animals *** p<0.0005 n = 8–9 per group.
(TIF)

S4 Fig A IFN-γ, B IL-6, C TNF-α and D IL-10 produced by re-stimulated MLN cells from naïve and *T*. *muris* infected APC^min/+^ mice at day 18 p.i. and naïve and *H*. *polygyrus* infected APC^min/+^ mice at day 28 p.i. E Number of CD4+CD25+FoxP3+ cells in MLN and spleen from naïve and *H*. *polygyrus* infected mice. Black bars denote naïve animals and grey bars denote infected animals * p<0.05, *** p<0.0005, $ significant difference between infected *T*. *muris* and infected *H*. *polygyrus* groups p<0.05. n = 7–9 per group.
(TIF)
