# Supplementary figures and images for: Chronic Trichuris muris infection causes neoplastic change in the intestine and exacerbates tumour formation in APC min/+ mice
Source: PLoS Negl Trop Dis. 2017 Jun 26;11(6):e0005708. doi: 10.1371/journal.pntd.0005708 (PMC5501682; doi:10.1371/journal.pntd.0005708)

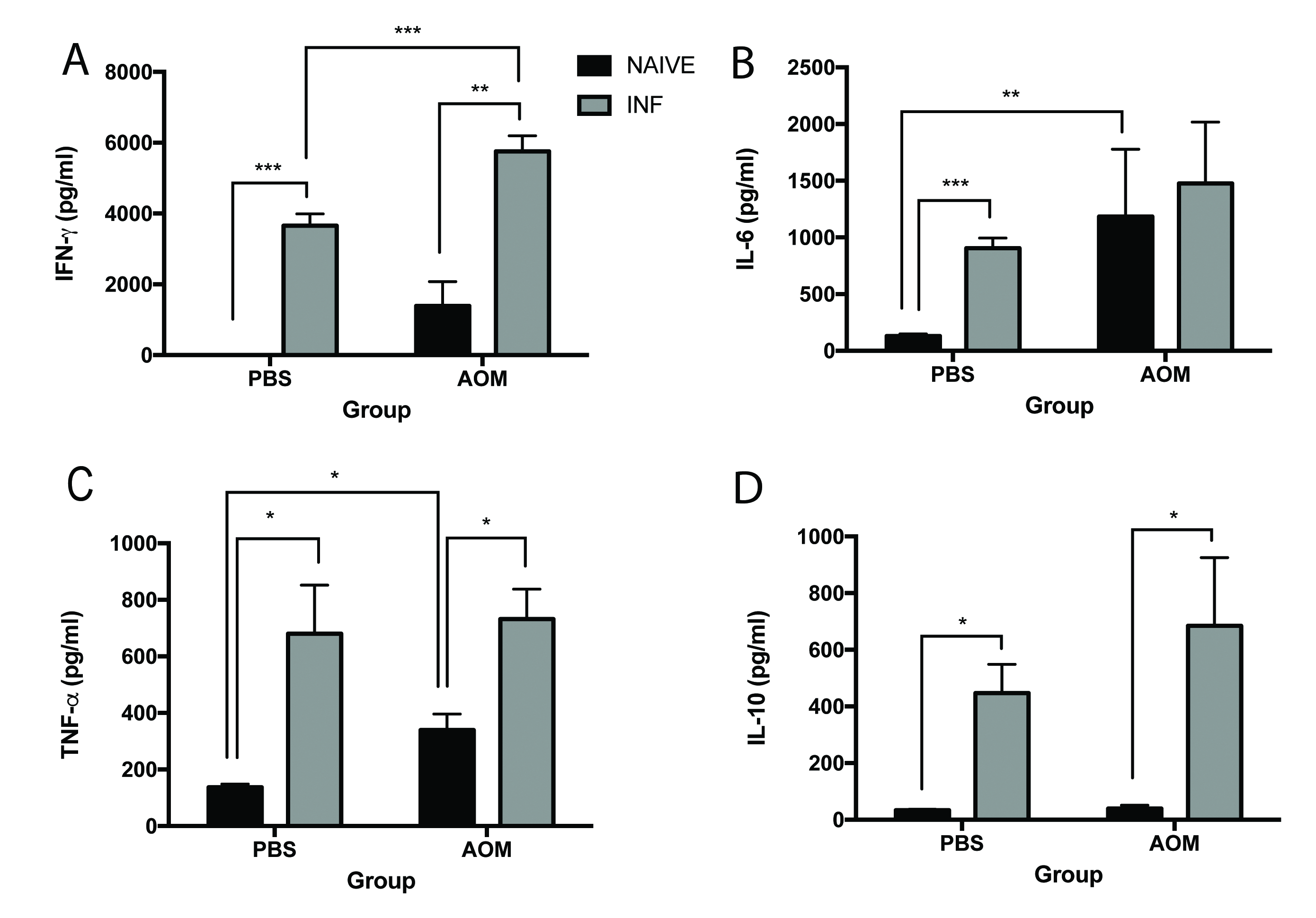

Supplement: S1 Fig — Black bars denote PBS treated animals and grey bars denote AOM treated animals. * p<0.05 ** p<0.005 ***p<0.0005 n = 4 per group. (TIF) [file pntd.0005708.s001.tif]

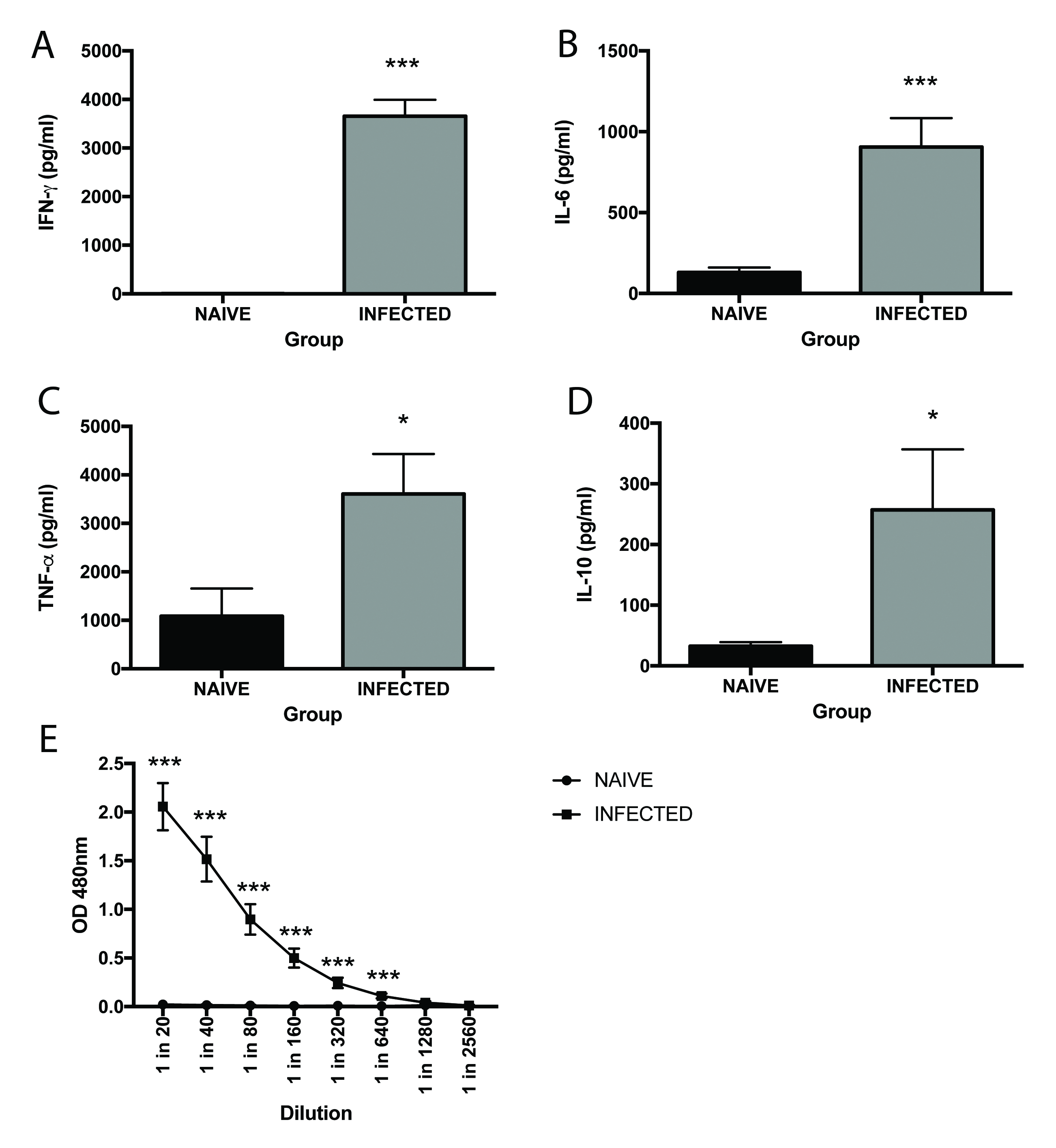

Supplement: S2 Fig — A IFN-γ, B IL-6, C TNF-α and D IL-10 produced by re-stimulated MLN cells from naïve and T. muris infected APCmin/+ mice at day 18 p.i. Black bars denote naïve animals and grey bars denote infected animals. E IgG2a production in naïve and T. muris infected APCmin/+ mice serum at day 42 p.i. * p<0.05, *** p<0.0005 n = 4 per group. (TIF) [file pntd.0005708.s002.tif]

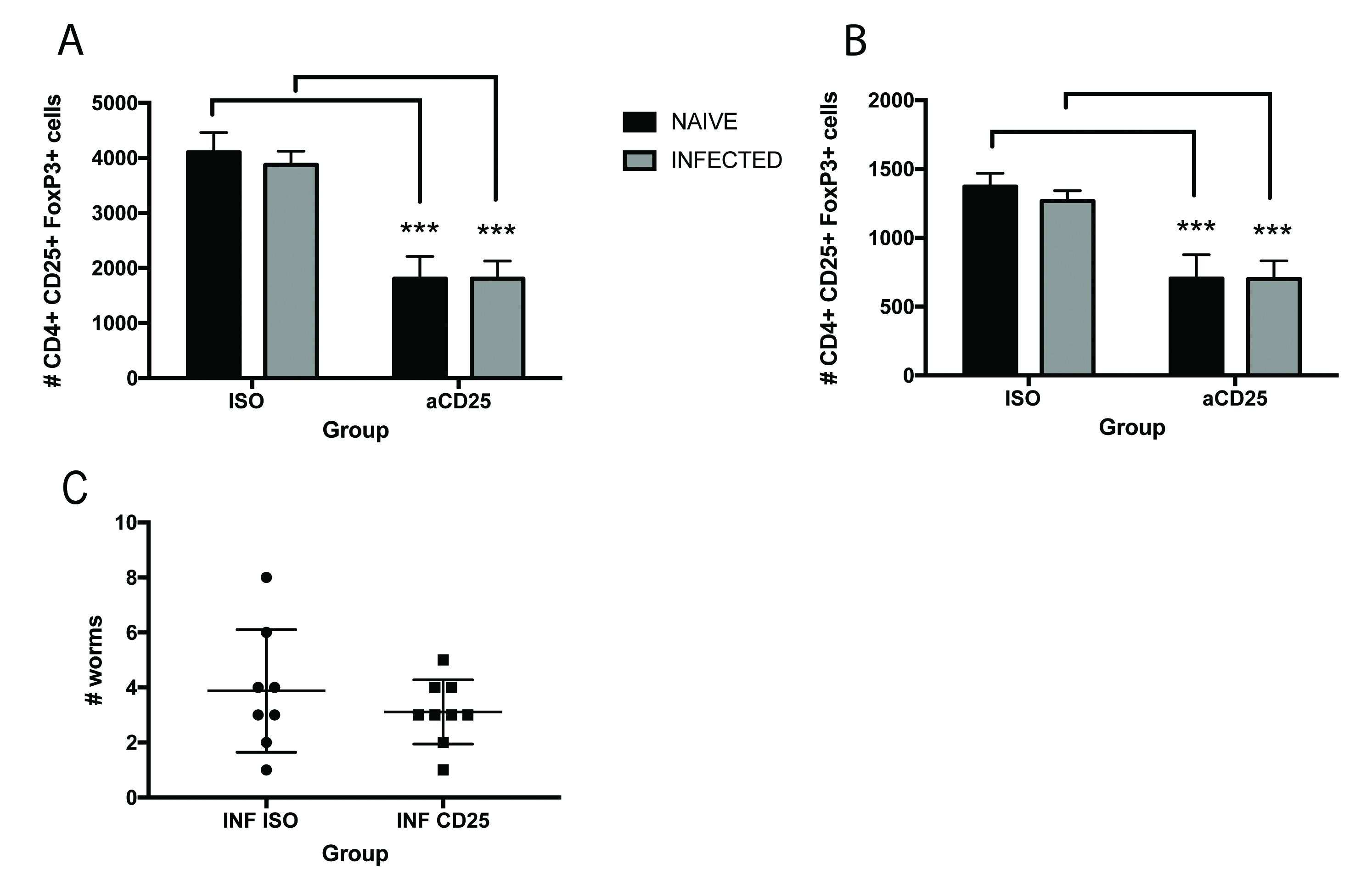

Supplement: S3 Fig — A Number of CD4+CD25+FoxP3+ cells in MLN and B Spleen from isotype and anti-CD25 treated naïve and T. muris infected APCmin/+ mice. C Worm burdens in isotype and anti-CD25 treated animals at day 18 p.i. Black bars denote naïve animals and grey bars denote infected animals *** p<0.0005 n = 8–9 per group. (TIF) [file pntd.0005708.s003.tif]

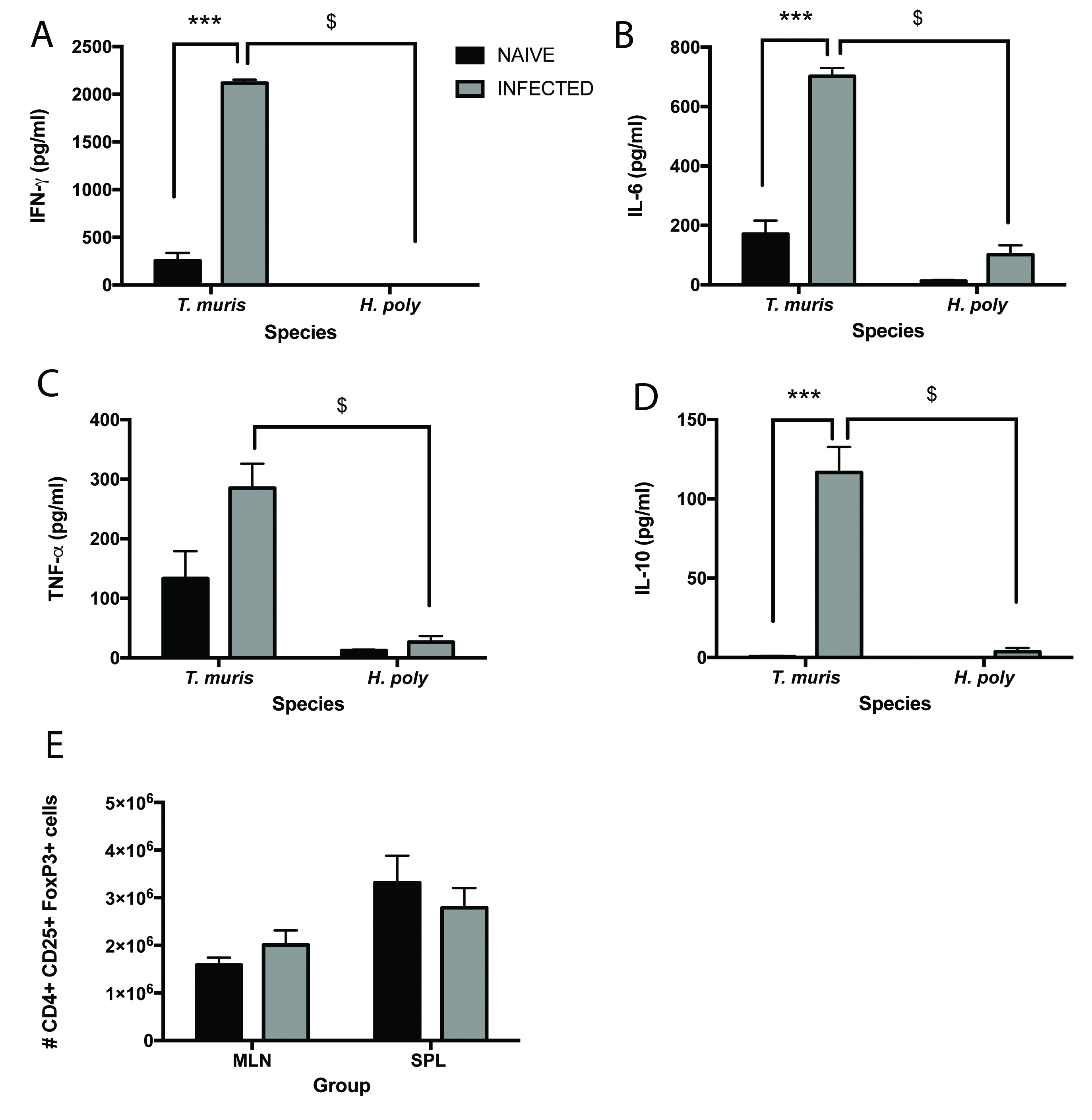

Supplement: S4 Fig — Black bars denote naïve animals and grey bars denote infected animals * p<0.05, *** p<0.0005, $ significant difference between infected T. muris and infected H. polygyrus groups p<0.05. n = 7–9 per group. (TIF) [file pntd.0005708.s004.tif]
